# Understanding fast-ion conduction in solid electrolytes

**DOI:** 10.1098/rsta.2019.0451

**Published:** 2021-11-29

**Authors:** Benjamin J. Morgan

**Affiliations:** Department of Chemistry, University of Bath, Claverton Down, Bath BA2 7AY, UK; The Faraday Institution, Quad One, Becquerel Avenue, Harwell Campus, Didcot OX11 0RA, UK

**Keywords:** fast-ion conduction, superionic conductivity, solid electrolytes, diffusion, solid-state ionics

## Abstract

The ability of some solid materials to exhibit exceptionally high ionic conductivities has been known since the observations of Michael Faraday in the nineteenth century (Faraday M. 1838 *Phil. Trans. R. Soc.* **90**), yet a detailed understanding of the atomic-scale physics that gives rise to this behaviour remains an open scientific question. This theme issue collects articles from researchers working on this question of understanding fast-ion conduction in solid electrolytes. The issue opens with two perspectives, both of which discuss concepts that have been proposed as schema for understanding fast-ion conduction. The first perspective presents an overview of a series of experimental NMR studies, and uses this to frame discussion of the roles of ion–ion interactions, crystallographic disorder, low-dimensionality of crystal structures, and fast interfacial diffusion in nanocomposite materials. The second perspective reviews computational studies of halides, oxides, sulfides and hydroborates, focussing on the concept of *frustration* and how this can manifest in different forms in various fast-ion conductors. The issue also includes five primary research articles, each of which presents a detailed analysis of the factors that affect microscopic ion-diffusion in specific fast-ion conducting solid electrolytes, including oxide-ion conductors ${\text{Gd}}_{2}{\text{Zr}}_{2}{\text{O}}_{7}$ and ${\text{Bi}}_{4}{\text{V}}_{2}{\text{O}}_{11}$, lithium-ion conductors ${\text{Li}}_{6}{\text{PS}}_{5}\text{Br}$ and ${\text{Li}}_{3}\text{OCl}$, and the prototypical fluoride-ion conductor β-${\mathrm{PbF}}_{2}$.

This article is part of the Theo Murphy meeting issue ‘Understanding fast-ion conduction in solid electrolytes’.

## 1. Introduction

Fast-ion–conducting solids are a intriguing class of materials that exhibit notably high ionic conductivities. This unusual property makes fast-ion conductors useful for applications such as all–solid-state batteries and fuel cells where they find use as solid electrolytes [1,2]. For these applications it is desirable to maximize ionic conductivity while preserving or optimizing for other application-specific properties, such as thermal or electrochemical stability, mechanical resilience, or sustainability. It is therefore useful to understand *why* certain materials exhibit particularly high ionic conductivities, while others, including materials that appear structurally or chemically similar, do not. Understanding the factors that promote fast-ion conduction in specific families of solid electrolytes can help direct the development of generalized ‘design principles’ that may then be used to design and synthesize new materials with improved ionic conductivities [3–8] or to identify completely new families of potential fast-ion conductors, through, for example, high-throughput computational screening [9–12]. The development of quantitative models of ion transport that can describe, and ideally also explain, the exceptional ionic conductivities of fast-ion conducting materials presents an additional intriguing challenge. In both cases, a necessary first step is to develop a detailed understanding of the chemical and structural factors that contribute to fast-ion conduction in specific materials.

Developing detailed quantitative descriptions of ion-transport in fast-ion conductors can often be challenging because of the tendency for these materials to exhibit complex ion-transport mechanisms. In conventional (non–fast-ion-conducting) solids, ionic transport is often well described as a sequence of discrete ‘hops’, where individual ions undergo stochastic moves between well-defined crystallographic sites [13–15]. In many fast-ion conductors, however, ion-transport proceeds via complex transport mechanisms. Ions may diffuse via highly concerted mechanisms in which groups of mobile ions undergo cooperative near-synchronous motion [16–21], or there may be strong coupling between the dynamics of the host-framework atoms and that of the mobile ions, with ion diffusion promoted by specific ‘breathing’ motions or by polyhedral rotations within the host framework [22–24]. The occurrence of complex conduction mechanisms in fast-ion conductors makes the analysis of experimental data and the development of explanatory or predictive theoretical models particularly challenging, especially in those materials where the microscopic mechanisms of ion transport have not yet been well characterized. The development of detailed quantitative descriptions of ion transport mechanisms in fast-ion conductors, and how these vary with changes in structure and chemistry, is therefore a vibrant and active area of research [5,16,18,24,25].

Within this context, there have been a number of recent studies that have presented new perspectives on the mechanistic origin of fast-ion conduction, or that have highlighted various concepts that can help to explain fast-ion conduction in specific materials. These factors include the role of various forms of inherent crystallographic disorder within the host-framework substructure [18,26–28] and the presence of occupational disorder of the mobile-ion species across available sites [29–31]. Other studies have analysed fast-ion conduction by considering ways in which a given system may be ‘frustrated’, where competing energetic considerations prevent the mobile ions from adopting a single ordered low-energy configuration, with these mobile ions instead sampling a large number of disordered configurations with similar energies [18,26,32–35]. In other materials, fast-ion conduction has been associated with specific host-framework dynamics, such as dynamical ‘breathing’ of diffusion-limiting bottlenecks [22], or polyanion rotations that may directly couple to the motion of the charge-carrying ions [24,36–38]. Investigations of the vibrational properties of fast-ion conductors have identified correlations between simple descriptors computed from the vibrational densities of states and ionic conductivities within families of solid electrolytes [9], while other studies have shown dramatic changes in the phonons of solid electrolytes as they undergo phase transitions from a low-temperature poorly conducting phase to a high-temperature ‘superionic’ phase [39–41]. Another interesting observation is that in a number of materials the mobile ions exhibit dynamical behaviours that are qualitatively similar to those in supercooled glass-forming liquids [16,18,21,31,42,43], with, for example, both classes of systems displaying ‘dynamic heterogeneity’, where some fraction of atoms participate in rapid highly correlated diffusion while other atoms are significantly less mobile [44,45].

These examples illustrate the breadth of ongoing research into understanding the mechanistic details and physical origins of fast-ion conduction in various families of solid electrolytes. These diverse phenomena provide ample scope for the interested researcher to find compelling scientific questions on which to focus. Research into fast-ion conductors, however, is often published and discussed in the context of specific families of solid electrolytes with distinct applications; e.g., focusing on fast-ion lithium conductors for solid-state lithium-ion batteries, or oxide-ion conductors for solid-oxide fuel cells. This theme issue instead brings together a series of perspectives and primary research articles to highlight some of the recurring themes and ideas across research sub-domains, with the intention of encouraging the cross-fertilization of ideas and perspectives within the broader research community.

## 2. Contributions to this issue

The first contributed article in this theme issue is ‘Fuzzy Logic: About the Origins of Fast Ion Dynamics in Crystalline Solids’ by Gombotz *et al.* [46]. This Perspective article presents a series of case studies where nuclear magnetic resonance (NMR) techniques have been used to probe the dynamics of mobile ions in a variety of fast-ion conducting solid electrolytes, and uses these to discuss a number of concepts and schema that can be used to rationalise the fast-ion conduction of specific materials. The authors discuss how site competition and Li–Li interactions produce concerted diffusion in ${\text{Li}}_{4+x}{\text{Ti}}_{5}{\text{O}}_{12}$ (LTO); the loss of site preference and associated Li disorder in Al-doped ${\text{Li}}_{7}{\text{La}}_{3}{\text{Zr}}_{2}{\text{O}}_{12}$ (LLZO) and ${\text{LiTi}}_{2}{\left({\text{PO}}_{4}\right)}_{3}$; the effect of cation disorder in $(\text{Ba},\text{Ca}){\text{F}}_{2}$ and related systems, and of anion disorder in the ${\text{Li}}_{6}{\text{PS}}_{5}\text{X}$ lithium argyrodites; the effect of low-dimensional diffusion pathways in layered materials; and the role of interfacial diffusion in two-phase nanocomposites.

The second contributed article is another Perspective: ‘Paradigms of Frustration in Superionic Solid Electrolytes’ by Wood *et al*. [47]. This article focuses on the concept of ‘frustration’ and how this can manifest in different ways in solid electrolytes, drawing from examples where atomistic simulations have been used to investigate this concept. The authors propose a classification of three types of frustration that each may facilitate fast-ion transport: *chemical frustration*, in which competition between different bonding interactions can produce a potential energy surface with multiple closely spaced local minima; *structural frustration*, in which interactions between mobile ions and the host framework prevent the mobile ions from relaxing into an ordered low-energy ground state; and *dynamical frustration*, which includes effects due to coupling between host-framework dynamics and mobile-ion dynamics.

The other articles in this theme issue present primary research into specific fast-ion conducting materials. The third and fourth articles both consider the role of host-framework disorder in promoting fast-ion conduction. The article by Annamareddy and Eapen, ‘Decoding Ionic Conductivity and Reordering in Cation-Disordered Pyrochlores’ [48] presents a study of how the configuration of host-framework cations in ${\text{Gd}}_{2}{\text{Zr}}_{2}{\text{O}}_{7}$ affects how mobile ${\text{O}}^{2-}$ ions are distributed over their available sites, and how this correlates with changes in their diffusion behaviour. The article by Sadowski and Albe, ‘Influence of ${\text{Br}}^{-}/{\text{S}}^{2-}$ Site-Exchange on Li Diffusion Mechanism in ${\text{Li}}_{6}{\text{PS}}_{5}\text{Br}$—A Computational Study’ [49] considers the effect of host-framework *anion* disorder in the lithium argyrodite ${\text{Li}}_{6}{\text{PS}}_{5}\text{Br}$, and presents a detailed mechanistic analysis of how the microscopic lithium-ion dynamics varies for different S/Br configurations. The authors also present a model to explain their results in which S/Br disorder can be understood in terms of separate ${\text{S}}_{\text{Br}}$ and ${\text{Br}}_{\text{S}}$ antisite defects and their net effect on their local lithium-ion distributions.

The fifth article, ‘Impacts of Vacancy-Induced Polarization and Distortion on Diffusion in Solid Electrolyte ${\text{Li}}_{3}\text{OCl}$’ by Mehmedovic *et al.* presents a detailed *ab initio* molecular dynamics (AIMD) study of the effect of off-stoichiometry, in the form of Li vacancies, in the antiperovksite solid electrolyte ${\text{Li}}_{3}\text{OCl}$. The authors show that the activation energy for diffusion varies with vacancy concentration, indicating a coupling between the hopping motions of individual lithium vacancies [50], and have proposed a model for $V_{\text{Li}}$–$V_{\text{Li}}$ interactions, mediated through local lattice strain and host-framework anion polarization, that promote correlated diffusion by lithium-vacancy pairs.

The sixth article, ‘Fast-Ion Conduction and Local Environments in BIMEVOX’ by Stroud *et al.* uses static density-functional theory (DFT) calculations and AIMD to study the potential energy landscape of the oxide-ion conductor ${\text{Bi}}_{4}{\text{V}}_{2}{\text{O}}_{11}$ as a function of O vacancy distribution [51]. One interesting aspect of this study is the combined use of static (geometry optimized) DFT data and dynamic AIMD simulations to derive ensemble averages for parameters such as the macroscopic diffusion coefficient by considering a thermodynamic sum over simulations that sample different O-vacancy configurations.

The seventh and final article in this special issue is ‘Cooperative Excitations in Superionic ${\text{PbF}}_{2}$’ by Mohn *et al.* [52]. This article presents another AIMD study, in this case used to analyse in detail the atomic-scale mechanism of collective diffusion processes in superionic β-PbF_2_, with a focus on how the formation of dynamical Frenkel pairs can trigger collective motion of string-like groups of migrating fluoride ions.

## 3. Conclusion

Fast-ion conducting solid electrolytes are a fascinating class of materials that often exhibit complex diffusion mechanisms. Modern experimental and computational techniques allow these diffusion mechanisms to be probed and analysed with increasing resolution and detail, allowing researchers to develop sophisticated descriptions of these mechanisms at the atomic scale, and how these vary with material parameters such as structure and chemical composition. For specific fast-ion conductors, understanding the microscopic origin of fast-ion conduction is often a compelling intellectual challenge in its own right. By going beyond merely cataloguing which material properties correlate with fast (or slow) ion-conduction, it may be possible to develop rational design principles for the optimization of specific families of fast-ion conductors with uses in targeted applications. Alongside this practical motivation for developing a *qualitative* understanding of the diffusion mechanisms in different fast-ion conducting solid electrolytes, the development of *quantitative* models of ion transport in fast-ion conductors continues to present a significant challenge. This special issue contains perspective articles that highlight some of the recurring themes and concepts within the field of understanding fast-ion conduction in solid electrolytes, as well as primary research articles detailing recent studies of ion-diffusion in a range of fast-ion conductors. It is this author’s expectation that future advances in the understanding of fast-ion conduction in solid electrolytes will be facilitated by increasing cross-fertilization of ideas and techniques between different materials-research communities, for example, from researchers working on materials for solid-state lithium-ion batteries, solid-oxide fuel cells, or hydrogen fuel cells, as well as communities focussed on more fundamental research into understanding complex atomic-scale dynamics, for example, in supercooled liquids.

## Data accessibility

This article has no additional data.
